# Editorial: Understanding the gene expression of β cell dysfunction in diabetes

**DOI:** 10.3389/fendo.2022.1069991

**Published:** 2022-11-11

**Authors:** Åke Sjöholm, John A. Corbett, Po Sing Leung, Bernard Portha

**Affiliations:** ^1^ Gävle Hospital, University of Gävle, Gävle, Sweden; ^2^ Department of Biochemistry, Medical College of Wisconsin, Milwaukee, WI, United States; ^3^ School of Biomedical Sciences, Faculty of Medicine, The Chinese University of Hong Kong, Shatin, China; ^4^ Laboratoire B2PE (Biologie et Pathologie du Pancréas Endocrine), Unité BFA (Biologie Fonctionnelle et Adaptive), CNRS UMR 8251, Université de Paris Cité, Paris, France

**Keywords:** diabetes, islet, gene expression, insulin secretion, GLP-1, beta cell (β cell)

The continued prevalence of diabetes mellitus imposes detrimental outcomes for millions across the world and contributes to put strain on health services. It is for such reasons that continued work into the understanding of the key components in the disease pathogenesis remains important. A central component in glucose homeostasis are pancreatic β cells, which function to secrete insulin. An understanding of the genes and their expression is critical to understanding how β cells function. Similarly uncovering the genetic and molecular mechanisms contributing to β cell loss and dysfunction is hoped to better our understanding of the pathogenesis of type 1 and type 2 diabetes. In addition to defining the candidate genes it is pertinent to understand their expression, how they are regulated and how their modulation may be associated with different outcomes in diabetes patients. Through this insight it may elucidate better treatment strategies to combat the disease progression.

Therefore, through this topic the gene expression underpinning β cell function and dysfunction has been addressed. In this thematic issue, eight papers dealing with genetic and epigenetic factors associated with β cell dysfunction, as well as the role of other key modulators and molecular mechanism in gene regulation, have been collected.

Professor Guy Rutter’s laboratory at Imperial College London contributes three papers:

Hu et al. describe functional genomics in β cells and review disease-associated genes. They also cover how recent advances in gene deletion and powerful genome editing technologies (such as CRISPR/Cas9) may be harnessed to advantage in diabetes research. Such tools provide valuable means of understanding β cell function, growth, and survival, how this is compromised in diabetes, as well as treating the disease.

The paper by López–Noriega and Rutter deals with long non-coding RNAs as key modulators of β cell mass and function. Since β cell mass is a major determinant of the amount of insulin that can be secreted by the pancreas, studies of this kind are paramount to advance our understanding of diabetes pathogenesis. They describe how long non-coding RNAs may be involved in controlling β cell proliferation and regeneration, as well as apoptosis.

In their third paper, Ghiasi and Rutter discuss the impact of unregulated transcript processing on β cell identity and function. They describe the possible role of regulated unproductive splicing and translation, a molecular mechanism involved in clearing non-functional transcript isoforms, whose dysregulation may be involved in β cell failure in diabetes.

Ramirez-Hernandez et al. describe the role of prolactin receptor and show that its deletion aggravates streptozotocin-induced diabetes. They report that this is caused by reduced islet density, β cell number, proliferation and viability, and suggest that prolactin-based agents may be useful as antidiabetic therapy. While previous reports have unequivocally shown that prolactin and the related growth hormone stimulate β cell proliferation and function *ex vivo*, one should remember that these hormones are generally considered diabetogenic in humans. However, it can easily be envisaged that prolactin receptor-agonistic agents may prove useful in promoting β cell number and boosting β cell replication and viability in islets prior to transplantation into patients.

Alhaidan et al. report on a case of diazoxide-responsive congenital hyperinsulinism with attendant hypoglycemia and attempt to functionally characterize a novel candidate gene. Through elaborate and careful molecular biology experiments, they managed to identify compound heterozygous variants in the adenylyl cyclase 7 gene, resulting in excessive glucose-stimulated insulin secretion causing hypoglycemia.

Ye et al. used bioinformatics analysis to screen and identify potential ferroptosis key gene in type 2 diabetic islet dysfunction. Ferroptosis is an iron-dependent type of programmed cell death that is characterized by the accumulation of lipid peroxides. After extensive screening using this powerful technique, they identified MGST1 (microsomal glutathione S-transferase 1) as an important ferroptosis gene in β cell failure in type 2 diabetes.

In the paper by Nord et al., mechanisms behind islet inflammation as a mediator of β cell damage and dysfunction in diabetes were addressed. More specifically, authors provide mechanistic information on how bromodomain and extraterminal domain (BET) inhibition can decrease islet inflammation and thereby mitigate the detrimental impact of β cells to proinflammatory cytokines. These results also support the potential therapeutic application of selective BET inhibitors in attenuating β cell inflammation, with obvious therapeutic implications in type 1 diabetes.

Finally, Zhang et al. investigated the correlation between transcription factor 7-like 2 (TCF7L2) gene polymorphisms and susceptibility of gestational diabetes. TCF7L2 encodes a transcription factor that is considered a critically important regulator of β cell function and also of GLP-1-producing enteroendocrine L-cells. It has garnered substantial interest also as a candidate gene in human type 2 diabetes. The authors’ findings suggest that four specific single-nucleotide TCF7L2 polymorphisms confer genetic susceptibility of gestational diabetes mellitus. These hypotheses-generating findings will prompt further studies for validation and identification of the underlying mechanisms.

## Author contributions

All authors listed have made a substantial, direct, and intellectual contribution to the work and approved it for publication.

## Conflict of interest

The authors declare that the research was conducted in the absence of any commercial or financial relationships that could be construed as a potential conflict of interest.

## Publisher’s note

All claims expressed in this article are solely those of the authors and do not necessarily represent those of their affiliated organizations, or those of the publisher, the editors and the reviewers. Any product that may be evaluated in this article, or claim that may be made by its manufacturer, is not guaranteed or endorsed by the publisher.

